# Final adult height in children with central precocious puberty – a retrospective study

**DOI:** 10.3389/fendo.2022.1008474

**Published:** 2022-12-02

**Authors:** Taja Knific, Melisa Lazarevič, Janez Žibert, Nika Obolnar, Nataša Aleksovska, Jasna Šuput Omladič, Tadej Battelino, Magdalena Avbelj Stefanija

**Affiliations:** ^1^ Faculty of Medicine, University of Ljubljana, Ljubljana, Slovenia; ^2^ Centre for Health Informatics and Statistics, Faculty of Health Sciences, University of Ljubljana, Ljubljana, Slovenia; ^3^ Department of Infectious Diseases, University Medical Center Ljubljana, Ljubljana, Slovenia; ^4^ Department of Vascular Surgery, Izola General Hospital, Izola, Slovenia; ^5^ Department of Pediatric Endocrinology, Diabetes and Metabolic Diseases, University Children’s Hospital, University Medical Centre Ljubljana, Ljubljana, Slovenia

**Keywords:** central precocious puberty, final adult height, gonadotropin-releasing hormone analog, triptorelin, growth, height prediction

## Abstract

**Background/Aims:**

Central precocious puberty (CPP) is due to premature activation of the hypothalamic-pituitary-gonadal axis. It predominantly affects girls. CPP leads to lower final height (FH), yet the treatment benefit in girls between 6 and 8 years is equivocal. Our main goal was to evaluate the effects of gonadotropin-releasing hormone analog (GnRHa) on FH and identify factors that predict FH.

**Methods:**

In a retrospective study, children with CPP (12 boys, 81 girls) that reached FH were included. Their clinical data at diagnosis and up to their final height was compared by descriptive statistics among idiopathic (iCPP) (n=68) and non-idiopathic CPP (nCPP) and between GnRHa treated (n=48) and untreated (n=15) girls with iCPP. The treatment effect of body weight (BW) adjusted GnRHa dosing was evaluated. Univariate linear regression and step-wise multivariable regression including 48 girls with iCPP treated with GnRHa were performed to identify predicting factors for FH.

**Results:**

Children with idiopathic CPP (iCPP) reached higher FH (p=0.002) than children with non-idiopathic CPP. After the diagnosis, the treated group gained 7.0 cm more than the untreated group. Yet, attributable to individualized decision-making, the FH in both groups was comparable (161.5 cm in treated, 161.0 cm in untreated girls with iCPP), although the onset of menarche was 2.5 years earlier among untreated girls. BW-adjusted dosing suppressed peak luteinizing hormone (LH) below 4.5 IU/L in 95% of children; however, bone age further advanced during therapy in 38% of patients. Predicting factors revealed by multivariable regression were bone age at diagnosis, BMI SDS at diagnosis, LH basal, age at start and cessation of treatment, predicted adult height and target height. (R2 = 0.72).

**Conclusion:**

Children with nCPP had worse FH outcome compared to iCPP despite similar CPP onset and therapeutic characteristics. Treatment by GnRHa using BW-adjusted dosing was effective in delaying menarche onset and reaching target height in girls with iCPP. Multiple factors affecting FH outcome indicated individualized decision-making regarding therapeutic intervention remains challenging. In the treated patients, among the factors that can be influenced, height at treatment cessation most significantly influenced the outcome.

## Introduction

Central precocious puberty (CPP) is defined as a premature activation of the hypothalamic-pituitary-gonadal (HPG) axis causing pulsatile hypothalamic secretion of gonadotropin-releasing hormone (GnRH) that consequently increases concentrations of luteinizing hormone (LH) and follicle-stimulating hormone (FSH). Pubertal LH and FSH secretion patterns increase the concentration of sex hormones that are then responsible for the early development of secondary sex characteristics (1, 2).

Based on six previous studies from Europe (3–6) and Korea (7, 8), the incidence ranges between 0.2 and 26.28 per 10 000 in girls and between 0.023 and 0.9 per 10 000 in boys. CPP occurs 10 times more commonly in girls than in boys (6). During recent decades, there has been a secular trend toward earlier onset of puberty, first reported in the United States in the 1990s, particularly in girls (3, 9). More recent European (3) and Korean (7, 8) studies also suggest an upward trend in the incidence of CPP. Numerous studies demonstrate that a secular trend for earlier menarche still occurs in the 21st century, not only in developing but also in developed countries, e.g., Canada, Denmark, Korea, and Spain. However, in the last couple of decades, in some countries, the trend for earlier menarche is slowing down or has stabilized, e.g., France, Germany, Greece, and the Netherlands (10). An important rise in the incidence of CPP observed in an Italian cohort of girls during the COVID-19 lock-down could serve as an example of a fast change in incidence upon environmental triggers (11). Such trends subject more and more children each year to the decision of whether to initiate treatment to stop the progression of puberty or not. It might as well be that the normal age at the beginning of puberty is decreasing (9, 12) and therefore physiologic puberty could be misdiagnosed as CPP, especially in girls (9).

Nevertheless, premature pubertal development carries certain physiologic and psychosocial risks (13). The most frequent long-term effect is lower final height (FH) due to estradiol’s effect on longitudinal bone growth (2). During early sexual maturation, low estradiol levels are responsible for a pubertal growth spurt, however, in late puberty, high concentrations of estradiol result in growth plate fusion and termination of bone growth (14). In addition, premature menarche is a risk factor for obesity, hypertension, type 2 diabetes mellitus, ischemic heart disease, stroke, cardiovascular mortality, and estrogen-dependent cancers (13). Some studies suggest that early stages of sexual maturation might be associated with hypersexual and delinquent behavior (13).

The mainstay of CPP treatment is a long-acting gonadotropin-releasing hormone analog (GnRHa) that suppresses the HPG axis by desensitizing the pituitary gonadotrophs (15). Its effects are regression or stabilization of pubertal symptoms and prolongation of growth period by lowering growth velocity to prepubertal values and decreasing bone-age advancement (16). There are various preparations of GnRHa available: intramuscular 4-week, 12-week, or 6-month depot forms, subcutaneous 1-year implant and 4-week, 12-week, and 6-month subcutaneous injection (17).

Efficacy of treatment is evaluated clinically by Tanner staging and linear growth assessment, radiologically by bone age maturation, and biochemically by LH measurements (13). If effective, regression of secondary sex characteristics, linear growth, and less advanced bone age are observed (16, 17).

While random ultrasensitive LH concentration can remain in the pubertal range despite puberty suppression, GnRH-stimulated LH peak inferior to 2.5-4.5 ml/U indicates a suppressed HPG axis (17). A recent study suggests first-void urinary LH concentration can also be useful in assessing puberty suppression, with a cut-off of 1.01 IU/L (18). In case treatment is found ineffective, the dose can be increased or the treatment can be administered more frequently (19).

The decision to suppress puberty is individualized and based on different clinical criteria. The most important indication is progressive precocious pubertal development and growth acceleration documented 3-6 months prior to GnRHa therapy (16). Treatment can be initiated without an observation period in the case of Tanner stage III and bone age advancement by more than 1 year or 2 SD (17), while bone age advancement less than 1 year doubts the diagnosis of precocious puberty. According to recommendations, treatment should be considered in girls before the age of 6 and boys before the age of 9 with progressive CPP, while in girls between 6 and 8 years the treatment decision is individualized (17). Cessation of treatment is also individualized in regards to chronological age, bone age, patient and family’s wishes, and synchronizing puberty with peers (20). Results were the most satisfactory when treatment was interrupted between 12 and 12.5 years of bone age in girls and between 13 and 13.5 years of bone age in boys (20).

Clinical decision to initiate treatment or not, particularly in children with later onset of CPP, is often difficult since factors that predict FH are not well defined. We aimed to evaluate the effect of the GnRH analog on FH and identify factors that predict FH in children with CPP to facilitate physicians’ decisions for treatment.

## Population and methods

A retrospective study included 93 children with CPP (12 boys and 81 girls) followed up at University Children’s Hospital in Ljubljana between 1994 and 2019, that reached their FH. Clinical data was collected from their medical records. Mandatory inclusion criteria were clinical signs of precocious puberty (progressive development of secondary sex characteristics including thelarche before the age of 8 or menarche before the age of 10 in girls and testicular volume larger than 3 ml before 9 years of age in boys) and biochemical signs of central puberty (LH basal > 0.2 IU/L and/or LH peak > 5 IU/L). Brain MRI was performed to identify potential organic cause of CPP in all 12 boys and in 32 girls (40%), of which 14 (17%) were older than 6 years. All 49 girls (60%) where we decided not to perform an MRI were older than 6 years and had slowly progressing central precocious puberty. In this manner, all cases with hypothalamic hamartoma were identified. In regards to etiology, children were divided into two groups. Children, who had no determined etiology of CPP (n=68) were considered idiopathic (iCPP). Children, who had an identified likely cause of CPP (n=25), such as structural or functional changes of the central nervous system potentially influencing the HPG axis, or genetic variants associated with precocious pubertal development, were considered non-idiopathic (nCPP). The potential causes of CPP in the nCPP group are described in **Table 1**. Clinical characteristics including FH between iCPP and nCPP groups were compared. Because of the large heterogeneity and additional short stature risks in several patients in the nCPP group and the small number of boys, further analyses were performed on iCPP girls only. We compared clinical characteristics between girls with iCPP treated with GnRHa triptorelin depot (n=48) and untreated girls with iCPP (n=15). The decision on who received treatment was made by trained pediatric endocrinologists based on individual characteristics and was not randomized. The main criteria to treat were the precocious start of puberty with lower predicted adult height and/or fast progressive puberty. The main criteria not to treat the child were slowly progressing puberty, nearly closed growth plates, close to normal height at diagnosis, severe comorbidity, or therapy refused by parents (rare). The main criteria to stop the treatment were height above 150 cm or age above 10 years in girls or bone age of 12 years in girls and 13 years in boys. Finally, to determine predicting factors for FH we conducted a univariable linear regression, a step-wise multivariable regression as well as ANCOVA analysis that included 48 girls with iCPP treated with triptorelin depot.

**Table 1:** Potential causes of central precocious puberty in the group considered non-idiopathic.

Potential cause of nCPP	Number of children	Percentage of children with nCPP
Hypothalamic hamartoma	4	16.0%
CNS infection	4	16.0%
CNS malignancy post-treatment	1	4.0%
Cerebral palsy	4	16.0%
Rathke cleft cyst	1	4.0%
Chiari 2 malformation, myelomeningocele	2	8.0%
Glioma due to neurofibromatosis, type 1	1	4.0%
Undefined structural change of pituitary	2	8.0%
Williams-Beuren syndrome	2	8.0%
Floating Harbor syndrome	1	4.0%
Down syndrome	1	4.0%
Pompe disease	1	4.0%
Congenital adrenal hyperplasia	1	4.0%

CNS, central nervous system.

The pubertal stage was evaluated by trained pediatric endocrinologists, testicular volume was estimated using Prader orchidometer. Tanner stage in boys was determined based on testicular volume using the following scale: T1 corresponded to less than 4 ml, T2 corresponded to 4-8 ml, T3 corresponded to 9-12 ml, T4 to 15-20 ml and T5 stage to more than 22 ml (21). Anthropometric measurements were performed by trained nurses using professional certified digital scales type Bolero (Arjo, Malmö, Sweden) and Digital Stadiometer 700-1600, QuickMedical^®^ (Warwick, RI, USA). GnRH stimulation test was performed using gonadorelin (Relefact LH-RH, Sanofi-Aventis, Germany) 100 µg/m^2^ body surface intravenously, blood samples were taken at 0, 20, 30, and 60 minutes and LH and FSH, as well as estradiol and testosterone, were measured by immunoassay using Immulite 2000.

Until July 2007 patients were treated with monthly injections of depot triporelin in a dose of 60 µg/kg of body weight, after this date all patient were receiving a 3 monthly triptorelin depot with the following doses: children with a body weight below 20 kg received 5.625 mg every 3 months, children with a body weight between 20 kg and 40 kg received 8.4375 mg every 3 months, and children with body weight above 40 kg received 11.25 mg every 3 months. Twelve patients (17%) were receiving monthly triptorelin throughout all therapy, 11 patients (15%) switched from monthly to 3-monthly triptorelin, and 48 patients (68%) were receiving 3-monthly triptorelin throughout all treatment. Children were clinically assessed for regression of pubertal signs and growth every 3 months, GnRH stimulation test was performed every 6 months in the first year of therapy and once yearly thereafter, and bone age was evaluated once yearly. Triptorelin doses were adjusted with weight gain and in case of insufficient HPG axis suppression.

Target height (TH) was calculated based on reported parental heights (mid-parental height + 6.5 [cm], boys; mid-parental height – 6.5 [cm], girls) (22). FH was defined as body height that increased by no more than 2 cm in 1 year or 1 cm in half a year. FH SDS was determined for 18 years since at that age FH is normally already reached. Predicted adult height (PAH) was calculated based on bone age, chronological age, height at diagnosis, and ethnicity (Central European) with BoneXpert Adult Height Predictor V3.0 (23). BMI SDS and body height SDS were determined based on British references from 1990 (24) with the LMS Growth program (25). Bone age and bone age SDS were determined from the left hand and wrist X-ray and based on the Greulich-Pyle method with either BoneXpert Adult Height Predictor V3.0 (23) for automated determination of bone age (42% of patients) or manually by experienced radiologists in children evaluated before the year of 2013 (58% of patients).

The study was approved by the Republic of Slovenia National Medical Ethics Committee (number 0120-141/2017-4, KME 48/04/17). Written informed consent was obtained before participation from adult patients or parents in the case of minors.

### Statistical analysis

Clinical features were compared between the groups using descriptive statistics. The results were presented as a median and interquartile range between the 1st and 3rd quartile since the data were non-normally distributed. The normality of data was tested with the Shapiro-Wilk test. Mann-Whitney U-test was used for continuous variables and Chi2 test or Fischer exact test for categorical variables. The limit for statistical significance was set to p <0,05. Univariable and step-wise multivariable linear regression were used to identify clinical features that influence FH in girls with CPP treated with GnRHa that have attained FH. The dependent variable in the regression analyses was the FH SDS. Independent variables that we tested were height SDS at diagnosis, bone age at diagnosis, bone age SDS at diagnosis, BMI SDS at diagnosis, basal and peak LH concentrations at diagnosis, age at menarche, Tanner stage, age at initiation of treatment, age at the cessation of treatment, duration of treatment, height at the cessation of treatment, height SDS at the cessation of treatment, TH SDS and PAH SDS. In addition, to determine the influence of Tanner stage on FH, we performed ANCOVA analysis by including the Tanner stage of puberty progression together with the significant variables from the stepwise model into the FH prediction. All statistical analyses were performed using R version 3.6 (The R Foundation for Statistical Computing, Vienna, Austria; https://www.R-project.org/).

## Results

### Final height in idiopathic versus non-idiopathic CPP group

The comparison between patients with iCPP and patients with nCPP is presented in **Table 2**. Girls in the iCPP group had breast development stages from B2 to B4 (31 girls (49%) at stage B2, 31 girls (49%) at stage B3, and 1 girl (2%) at stage B4) and boys had testicular volume ranging from 4 ml (4 boys) to 10ml (1 boy). In the nCPP girls, breasts were at stage B2 in 11 girls (61%) and at stage B3 in 7 girls (39%). Several nCPP boys had advanced puberty, testicular volume ranged from 4 ml to 22 ml (4 ml in 2 boys, 5 ml in 1 boy, 10 ml in 1 boy, 15 ml in 1 boy, 20 ml in 1 boy, and 22 ml in 1 boy). There was no statistically significant difference in stages of puberty between the two groups.

**Table 2:** Comparison between idiopathic and non-idiopathic CPP.

	All children with CPPN=93	iCPP groupN=68	nCPP groupN=25	p-value
Age at diagnosis [years]	7.83 [6.99;8.67]	7.84 [7.04;8.60]	7.76 [6.87;8.86]	0.778
Lag from onset of secondary sex characteristics to diagnosis [years]	0.73 [0.36;1.28]	0.80 [0.40;1.29]	0.57 [0.27;1.16]	0.213
Sex (M/F)	12/81	5/63	7/18	0.014
Height SDS at diagnosis	1.32 [0.07;2.32]	1.41 [0.50;2.32]	0.55 [-0.87;2.85]	0.167
BMI SDS at diagnosis	1.28 [0.24;2.00]	1.06 [0.26;1.92]	1.39 [0.20;2.34]	0.202
Bone age SDS at diagnosis	2.19 [1.45;3.47]	2.20 [1.57;3.39]	2.16 [1.07;3.59]	0.959
LH basal [IU/L]	0.33 [0.16;1.04]	0.29 [0.15;0.75]	1.00 [0.30;1.90]	0.006
LH peak [IU/L]	14.7 [7.21;31.8]	9.16 [6.34;22.3]	35.0 [17.5;45.0]	0.001
Tanner stage at diagnosis T2 T3 T4 T5	49 (52.7%) 40 (43.0%) 3 (3.23%) 1 (1.08%)	35 (51.5%) 32 (47.1%) 1 (1.47%) 0 (0.00%)	14 (56.0%) 8 (32.0%) 2 (8.00%) 1 (4.00%)	0.087
Treatment with GnRHa (Yes/No)	71/22	52/16	19/6	1.000
Age at initiation of treatment with GnRHa [years]	7.85 [6.99;8.59]	7.79 [7.00;8.58]	8.18 [6.73;8.66]	0.825
Age at cessation of treatment with GnRHa [years]	10.1 [9.73;10.5]	10.0 [9.72;10.4]	10.3 [9.80;10.9]	0.392
Duration of treatment with GnRHa [months]	24.4 [4.83;32.9]	23.6 [4.83;31.1]	25.6 [8.43;37.5]	0.450
FH SDS	-0.51 [-1.25;0.57]	-0.30 [-1.09;0.73]	-1.01 [-3.24;-0.43]	0.002
TH SDS	-0.01 [-0.59;0.57]	-0.24 [-0.84;0.55]	0.24 [-0.05;0.73]	0.030
PAH SDS	0.12 [-0.67;0.95]	0.21 [-0.37;0.93]	-0.54 [-1.66;0.91]	0.057
SDS FH- SDS TH	-0.55 [-1.16;0.27]	-0.25 [-0.89;0.53]	-1.31 [-2.73;-0.72]	<0.001
SDS FH- SDS PAH	-0.57 [-1.37;0.07]	-0.52 [-1.16;0.15]	-1.08 [-2.20;-0.10]	0.044

The results are presented as a median and interquartile range between 1st and 3rd quartile. M, male; F, female; LH luteinizing hormone; SDS – standard deviation score; FH, final height; TH, target height; PAH, predicted adult height.

Otherwise, the children in the two groups had similar clinical features at diagnosis and comparable GnRHa treatment characteristics. The two groups differed in LH peak value which was higher in the nCPP group, indicating children with nCPP likely had more advanced puberty at diagnosis, which is supported also by more advanced Tanner stage in nCPP boys. The nCPP group also included significantly more boys. The children with iCPP reached significantly higher FH than children with nCPP (p=0.002). Negative FH SDS in both groups indicated lower FH in children with CPP as compared to the general population. Yet, considering the heights near FH were included, the difference from the general population in the iCPP group was very small. On the other hand, the nCPP group was etiologically very heterogeneous (**Table 1**), with children having conditions that could independently participate to short stature, including genetic disorders. One child with panhypopituitarism was receiving also growth hormone and L-thyroxin supplementation. Additionally, a child with Floating-Harbor syndrome was receiving growth hormone for short stature after being born with low birth weight.

The difference between FH and TH indicated the difference between the actual attained height and growth potential the child had. The median was negative in both groups which indicated that children with CPP were below their target height. The difference between two groups was significant: FH in children with iCPP was only 1,5 cm below TH while in the nCPP group it was considerably lower at 9 cm below TH. Taking into consideration that TH reported from parental heights is often overestimated (26), it is not unlikely that children with iCPP actually reached their TH in contrast with children with nCPP that were further below their TH. The median difference between FH and PAH was also negative, meaning neither group attained its predicted height.

The median age at onset of puberty as reported by the parents was 7 years in both groups (iCPP: Q1 = 6.00, Q3 = 7.83; nCPP: Q1 = 5.00, Q3 = 7.75). Median lag from onset of symptoms until diagnosis was not significantly different between two groups: 0.80 years (Q1 = 0.50, Q3 = 1.29) in the iCPP group and 0.57 years (Q1 = 0.27, Q3 = 1.16) in the nCPP group.

### Final height in treated versus untreated group

The comparison between girls with iCPP treated with GnRHa and girls with iCPP without any intervention is presented in **Table 3**.

**Table 3:** Comparison between treated and untreated girls with iCPP.

	All children reached FH	Treated with GnRHa	Not treated	p-value
	N=63	N=48	N=15	
Age at diagnosis	7.85 [7.02;8.57]	7.55 [6.41;8.14]	8.78 [8.44;8.97]	<0.001
Height SDS at diagnosis	1.40 [0.50;2.31]	1.37 [0.49;2.25]	1.66 [1.10;2.32]	0.439
Bone age SDS at diagnosis	2.19 [1.48;3.34]	1.97 [1.29;3.32]	2.76 [1.99;3.22]	0.246
Age at menarche [years]	11.0 [10.5;12.0]	11.5 [11.0;12.0]	9.00 [9.00;9.75]	<0.001
Tanner stage at diagnosis T2 T3 T4	31 (49.2%) 31 (49.2%) 1 (1.59%)	25 (52.1%) 22 (45.8%) 1 (2.08%)	6 (40.0%) 9 (60.0%) 0 (0.00%)	0.660
**Final height**				
FH SDS	-0.34 [-1.09;0.73]	-0.38 [-1.10;0.73]	-0.09 [-0.92;0.40]	0.955
FH-TH [cm]	-1.50 [-5.50;2.30]	-1.50 [-6.25;3.20]	-2.00 [-3.75;0.75]	0.980
FH-PAH [cm]	-3.50 [-7.10;0.40]	-3.50 [-7.25;-0.50]	-1.95 [-5.23;1.35]	0.395
TH SDS	-0.34 [-0.84;0.57]	-0.34 [-0.82;0.57]	-0.18 [-1.05;0.61]	0.947
PAH SDS	0.20 [-0.41;0.85]	0.17 [-0.42;0.97]	0.29 [-0.08;0.75]	0.867
FH SDS – TH SDS	-0.25 [-0.91;0.58]	-0.24 [-1.03;0.59]	-0.33 [-0.62;0.12]	0.940
FH SDS – PAH SDS	-0.56 [-1.16;0.07]	-0.56 [-1.18;-0.08]	-0.32 [-0.86;0.22]	0.456
FH- height at diagnosis [cm]	28.4 [23.2;34.3]	31.0 [25.8;35.1]	24.0 [20.9;27.0]	0.005

The results are presented as a median and interquartile range between 1st and 3rd quartile. M,male; F,female; LH, luteinizing hormone SDS – standard deviation score; FH, final height; TH, target height; PAH, predicted adult height.

There was no significant difference in the FH between the treated and untreated girls. However, the study was retrospective and the decision to initiate treatment was individualized and not randomized. Nevertheless, the treated group gained 31.0 cm (Q1 = 5.8; Q3 = 35.1) since CPP diagnosis up to the FH as compared to 24.0 cm (Q1 = 20.9; Q3 = 27.0) in the untreated group, while at the diagnosis the treated group was younger. There was a trend towards the lower height and bone age SDS at diagnosis in the treated group, yet the difference was not significant, which could be due to the small groups. Finally, age at menarche was below the normal range in the untreated girls, as an irrevocable sign of precocious pubertal development. Final height SDS was negative in both groups, indicating the mean FH of girls with iCPP was lower compared to their peers. Moreover, neither group exceeded their predicted height. The difference between FH and TH was only 1,5 cm in the treated group and 2 cm in the untreatead group, hence, considering that TH is often overestimated (26) there is a possibility that the children actually reached their TH.

### Evaluation of efficacy of treatment

We evaluated the effect of treatment by GnRHa by measurements of LH (shown in **Table 4**) and by evaluation of growth and bone age advancement (shown in **Table 5**). The median LH peak was in the prepubertal range after 6 months of treatment. The HPG axis was sufficiently suppressed in 89% of patients considering the low cut-off LH peak values [2.5 IU/L (27)] and in 95% of patients if we consider higher peak cut-off (LH 4.5 IU/L (28)). The median basal value LH remained elevated after 6 months; however, random ultrasensitive LH levels can remain high despite puberty suppression and are not a good measurement to asses treatment effectiveness (29). Height SDS advanced in the first year of treatment and decreased in the second year. Similarly, growth velocity remained increased in the first year of treatment and slowed down to the prepubertal growth velocity in the second year. The median BMI SDS increased in the first as well as in the second year. The median bone age was more advanced 1 year after diagnosis. Bone age SDS decreased in 50% of patients, remained the same (<0.10 change in SDS) in 11%, and increased in 39% after 1 year of treatment. The dose of GnRHa was adjusted according to weight gain and in 2 patients due to insufficient suppression of the HPG axis according to the GnRH stimulation test.

**Table 4:** Evaluation of HPG axis suppression by GnRH stimulation test.

	At diagnosis	After 6 months
**LH peak**	16.5 [7.26;30.6]	1.46 [1.02;1.89]
LH peak >2.5	98%	11%
LH peak >2.5	2%	89%
LH peak >4.5	92%	5%
LH peak <4.5	8%	95%
**LH basal**	0.30 [0.16;0.76]	0.45 [0.31;0.64]

The results are presented as a median and interquartile range between 1st and 3rd quartile. LH, luteinizing hormone.

**Table 5:** Evaluation of anthropometric changes and bone age 1 year and 2 years after treatment.

	At diagnosis	1 year	2 years
Height SDS	1.11 [0.02;2.24]	1.54 [0.35;2.58]	1.25 [0.33;2.24]
BMI SDS	0.9 [0.12;1.92]	1.34 [0.65;1.89]	1.47 [0.74;1.91]
Bone age SDS	1.96 [1.12;3.43]	2.37 [1.49;3.70]	
Yearly height SDS difference		0.21 [-0.05;0.36]	-0.20 [-0.33;0.05]
Yearly growth velocity [cm/year]”		6.89 [6.07;7.91]	5.26 [3.94;6.05]
Difference in growth velocity between 1st and 2nd year [cm/year]			-2.11 [-3.55;-0.50]
Bone age SDS difference in 1 year		0.14 [-0.32;1.40]	

The results are presented as a median and interquartile range between 1st and 3rd quartile. Abbreviations: SDS- standard deviation score, BMI- body mass index

### Predictors of final height in iCPP girls treated with GnRHa

Predicting factors for FH revealed by step-wise multivariable linear regression analysis were bone age at diagnosis, BMI SDS at diagnosis, LH basal, age at the initiation of treatment, age at the cessation of treatment, height at the cessation of treatment, TH SDS, and PAH SDS. Our model explained 72% of final height variance in girls with iCPP treated with GnRHa (R^2^ = 0.72) (**Table 6**).

**Table 6:** Multivariable analysis of variables significantly associated with final height SDS of 48 girls with iCPP treated with GnRHa.

Independent variables	β -Coefficient	p-value
Height SDS at diagnosis	-0.881	0.091
Bone age at diagnosis [years]	0.382	0.002
BMI SDS at diagnosis	-0.200	0.036
LH basal [IU/L]	-0.345	0.024
Age at initiation of treatment with GnRHa [years]	-0.409	0.006
Age at cessation of treatment with GnRHa [years]	0.473	0.011
TH SDS	0.427	0.006
PAH SDS	0.675	<0.001

SDS, standard deviation score; BMI, body mass index; LH, luteinizing hormone; GnRHa gonadotropin-releasing hormone analog; TH, target height; PAH, predicted adult height.

**Figure 1:**
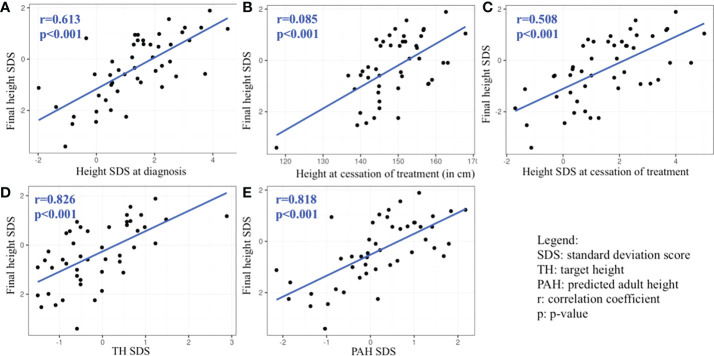
Graphs **(A–E)**: Statistically significant predictors associated with final height SDS revealed by univariable analysis.

The univariable linear regression analysis revealed that FH was positively and significantly associated with height SDS at diagnosis, height at the cessation of treatment, height SDS at the cessation of treatment, TH SDS, and PAH SDS. FH was not significantly negatively associated with any of the variables we tested (**Table 7** and **Figure 1**).

**Table 7:** Univariable analysis of predictors associated with final height SDS in 48 girls treated with GnRHa.

Predictors	Correlation coefficient (r)	p-value
Height SDS at diagnosis	0.613	<0.001
Bone age at diagnosis [years]	0.0234	0.808
Bone age SDS at diagnosis	0.116	0.200
BMI SDS at diagnosis	-0.0136	0.757
LH basal [IU/L]	0.146	0.455
LH peak [IU/L]	0.0091	0.532
Tanner stage	0.3142	0.386
Age at menarche [years]	0.130	0.473
Age at initiation of treatment with GnRHa [years]	-0.154	0.116
Age at cessation of treatment with GnRHa [years]	-0.201	0.294
Duration of treatment [months]	0.010	0.308
Height at cessation of treatment [cm]	0.084	<0.001
Height SDS at cessation of treatment	0.508	<0.001
TH SDS	0.826	<0.001
PAH SDS	0.818	<0.001

SDS, standard deviation score; BMI, body mass index; LH, luteinizing hormone; GnRHa gonadotropin-releasing hormone analog; TH, target height; PAH, predicted adult height.

The ANCOVA analysis including Tanner stage of puberty progression together with the significant variables from stepwise model into FH prediction showed no significant influence of the Tanner stages on the FH with a p-value = 0.631 (F=0.2355). Nevertheless, only one girl had at diagnosis puberty advancement at stage B4, while the others had either B2 or B3 stages (**Table 3**). Our results are therefore not applicable for wider differences in Tanner stages.

## Discussion

Reaching the genetic height potential or at least achieving a normal adult height is one of the major goals in decision-making when confronted with a child with precocious puberty. Particularly in girls, intervention usually interferes with their growth spurt which generally occurs during early puberty. The benefit of puberty suppression is unequivocal in girls affected by CPP before 6 years of age and in boys affected by CPP before 9 years of age. On the other hand, the results of treatment in girls affected by CPP between 6 and 8 years, which are by far the commonest cases of CPP in clinical practice, are variable, desiring individualized and challenging managing decisions. With our study, we aimed to critically address the decision-making and treatment regimen by analyzing FH and factors influencing FH in different groups of children with CPP.

A comparison of children with idiopathic and non-idiopathic CPP suggested the non-idiopathic group had a significantly worse FH prognosis despite similar onset and therapeutic characteristics which is comparable to a study in Spanish girls with the organic cause of CPP (30). Another study following patients with CPP due to treatment of tumors near the hypothalamus suggests that height potential is significantly compromised in such conditions (31). Nonetheless, Ramos et al. demonstrated that children with CPP due to hypothalamic hamartoma reach normal or near normal FH (32). Moreover, PAH SDS in our study did not differ between the two groups, suggesting lower FH in the nCPP group might not be due to a more rapid progression of puberty, but rather due to a hypothalamic dysfunction following CNS lesions or their treatment, CNS infection or hydrocephalus following myelomeningocele, as previously suggested (33). It should also be noted, that our cohort is etiologically heterogeneous, including organic and genetic causes of CPP. Individual nCPP patients had genetic syndromes associated simultaneously with CPP and short stature, such as Williams-Beuren syndrome (34) and Floating Harbor syndrome (35). A girl with Down syndrome and CPP was also included in the nCPP group, though Down syndrome is more often associated with peripheral precocious puberty (36).

Similar to other studies (37), children with CPP from our study also reached lower FH compared to the general population. The difference between FH and TH for the whole cohort was at 1.5 cm, whereas, in other studies, the difference ranges between 7 cm below and 1 cm above TH (38). There was a striking difference in this parameter between the iCPP group where FH was 1.5 cm below TH and the nCPP group with a difference of 9 cm. While interestingly, the difference between FH and TH was insignificant comparing the treated with untreated iCPP girls. Considering that TH was calculated from the heights reported by the parents, which are notoriously overestimated (26), it is almost certain that the actual TH is lower than the calculated TH. And also, the near FH was measured, thus it cannot be excluded that these children would gain at least 1 cm in the following years, therefore the actual FH might be equal or even greater than the mid-parental height in the group of iCPP.

Evaluation of the impact of GnRHa on FH is difficult since no control studies exist and we have to rely on retrospective clinical data when comparing treated with untreated children. The benefit of GnRHa on FH depends on the age at initiation of treatment. For girls, height gain ranges from 2 to 10 cm (39). Girls benefit most when treatment is initiated before 6 years of age (40), between 6 and 8 years outcomes are variable and after 8 years there is usually no increase in adult height (41). There are fewer studies with smaller sample sizes on the effect of GnRHa on FH in boys, however, existing studies suggest that boys do benefit from treatment and PAH improves throughout treatment (42). Doses of GnRHa for optimal HPG suppression are not well defined and they vary in different countries. Lower doses (3.75 mg/28 days, 80- 120 µ/kg/28 days) are preferred in Europe and Asia, whereas in the US doses are higher (7.5-15 mg/28 days, 200-300 µ/kg/28 days) (43). A Japanese study concluded that the minimum effective suppressive dose is already 30 µg/kg (44). A Turkish study suggests that higher doses may be required for cases at advanced stages and increased body weight (>36.2kg) or BMI (>1.64 SDS) (43). Of note, the concerns of over suppression are suppression of growth, a possible transient decrease in bone mineral density and mineral accrual, as well as the cost of treatment (43). In our study, the dose was titrated according to the body weight. While peak LH was sufficiently suppressed in the majority of patients and only in two patients (5%) the dose was increased due to insufficient LH suppression, bone age kept advancing in 38% of patients, which could have influenced their FH achievement. Nevertheless, the body weight-based adjusted dose of triptorelin in our study showed efficiency in attaining normal FH and TH.

The effect of GnRHa treatment on an increase in BMI is not clear. While some studies (45, 46) demonstrate an increase in BMI during treatment, others do not report any influence on weight gain during therapy (47–49). We observed an increase in BMI over the two years of treatment as well as an increased BMI already at diagnosis; this observation is comparable to an Italian study (50). Increased BMI at diagnosis is a common finding in iCPP in girls. For reawakening of the GnRH pulse generator to signal the onset of puberty, the female must sense she is equipped with enough energy to sustain the increased metabolic demands of puberty, especially of menarche (51). It is known that a critical threshold of body fat is necessary for sexual maturation (52). Accordingly, most girls who enter puberty at an early age have an above average BMI z-score (53).

Even though our study found no difference in FH SDS between the treated and untreated patients, it is important to note that neither group’s FH deviated significantly from normal adult height. The decision for no treatment in an individual with CPP was always taken after a thorough clinical evaluation of the expected potential benefits of the therapy in regards to the progression of puberty, onset of menarche, age at diagnosis, PAH, and height at diagnosis. Later onset of menarche in the treated group was expected and attributed to the efficacy of GnRHa to postpone puberty, which was similarly observed in an Italian study of girls with iCPP (54). Excess height gain of 7 cm more in the treated group is also a positive indicator of efficacy which was also well comparable to other similar studies (39). While the treated girls were younger, there was also a trend toward lower height and bone age SDS at diagnosis in the treated girls.

A comparison between height outcomes in girls treated with GnRH from different studies is shown in **Table 8**. We remark that chronological age at diagnosis and bone age at diagnosis from similar studies are comparable to ours. FH in our study was satisfactory and even slightly higher compared to 12 other studies shown in **Table 8**. Nonetheless, we did not take into consideration different ethnic backgrounds, which could be reflected in lower TH in some studies. The difference between FH and TH varied, meaning some cohorts exceeded and some did not reach their target height. We consider our treatment regimen effective in attaining normal adult height and reaching a genetic potential regarding FH.

**Table 8:** Comparison of height outcomes in girls treated with GnRHa from different studies.

	Treatment	BW dependant dose	N	CA at diagnosis [years]	BA at diagnosis[years]	CA at cessation of treatment[years]	TH[cm]	PAH[cm]	FH[cm]	FH-TH[cm]	FH-PAH[cm]
Our study	Until July 2007: Depot Triptorelin 60µg/kg of BW every month. From July 2007 onwards: Triptorelin 5.625mg for <20kg, 8.4375mg for 20-40kg, 11.25mg for >40 kg every 3 months	Yes	48	7.3+1.8	8.2 ± 1.9	10.1 ± 0.9	164.8 ± 7.0	162.7 ± 5.6	161.5 ± 7.5	-1.4	-3.5
Oostdijk et al, 1996 (55)	Triptorelin 3.75mg/4 weeks	No	31	7.7	10.8	11.1	168.7 ± 6.4	156.2 ± 7.4	161.6 ± 7.0	-7.1	3.4
Carel et al, 1999 (45)	Triptorelin 3.75 mg/4 weeks for >20kg, 1.87 mg/4 weeks for <20kg	Yes	58	7.5 ± 1.3	10.1 ± 1.5	11.0 ± 1.1		155.2 ± 4.7	158.5 ± 5.3		3.2
Heger et al, 1999 (56)	Triptorelin 75μg/kg BW/30 ± 2 days	Yes	50	6.2 ± 2.0	9.3 ± 2.5	11.0 ± 1.1	163.6 ± 6.2	154.0 ± 9.6	160.6 ± 8.0	-2	5.7
Leger et al, 2000 (57)	Triptorelin 3.75mg/4 weeks	No	9	8.7 ± 0.4	11.1 ± 0.4	10.8 ± 0.6	159.8 ± 4.6	155.3 ± 5.6	160.2 ± 6.7	0.4	4.9
Partsch et al, 2000 (58)	Triptorelin 75 μg/kg/4 weeks	Yes	52	6.2 ± 0.3	9.3 ± 0.3	11.1 ± 1.1	164	154.9 ± 9.6	160.6 ± 8.0	-3.4	5.7
Adan et al, 2002 (59)	Triptorelin 3.75 mg/25 days for >20kg, 1.87 mg/4 weeks for <20kg	Yes	43	7.9 ± 1.3	10.3 ± 1.3	10.8 ± 0.7	161.2 ± 4.6	156.0 ± 7.8	159.5 ± 5.3	-1.7	3.5
Lazar et al, 2007 (40)	Depot Triptorelin 1.5-3.0 μg/kg/release per day administered every 4 weeks, maximal dose 3.75	Yes	60	<6y: 6.4 ± 1.2	8.9	11.3 ± 0.4	159.3 ± 5.0	154.6 ± 6.6	162.8 ± 5.0	3.1	8.2
				>6y: 7.5 ± 0.6	10	11.3 ± 0.3	157.8 ± 5.2	153.7 ± 6.7	157.9 ± 5.1	4.2	0.1
Pasquino et al, 2008 (60)	Triptorelin 100-120 μg/kg/21-25 days	Yes	87	8.4 ± 1.5	11.1 ± 1.6	12.6 ± 1.0	157.6 ± 4.7	154.2 ± 5.3	159.8 ± 5.3	2.2	5.6
Lee et al, 2011 (61)	Leuprolide acetate 300 μg/kg/4 weeks	Yes	29	7.3 ± 1.9	10.2 ± 2.13		163.8	157.4	162.5	-1.3	5.1
Poomthavorn et al, 2011 (62)		No	47	8.5 ± 1.0	11.1 ± 1.7	11.8 ± 1.0	155.8 ± 4.1	155.3 ± 6.7	158.6 ± 5.2	2.8	3.3
Gillis et al, 2013 (63)	Triptorelin 75–150 μg/kg/4 weeks	No	23	8.4 ± 0.3	10.4 ± 0.4	11.7	160.8 ± 0.8	155.2 ± 1.9	157.9 ± 1.7	-0.9	2.7
Jung et al, 2014 (64)	Triptorelin 75–150 μg/kg/4 weeks	Yes	59	8.7 ± 0.8	10.2 ± 1.6	10.6 ± 0.8	159.9 ± 3.5	156.6 ± 4.0	160.4 ± 4.2	0.5	3.8
Liang et al, 2015 (65)	Triptorelin 3.75mg/6 weeks	No	17	8.1 ± 0.2	9.2 ± 0.3		158.3 ± 0.9	161.6 ± 0.9	159.8 ± 1.2	1.5	-1.8
Bertelloni et al, 2015 (66)	Triptorelin 11.25 mg/3 months	No	12	7.9 ± 0.6	10.6 ± 0.9		159.7 ± 3.8	155.0 ± 3.5	157.1 ± 4.9	-2.6	2.1

Values are presented as average and standard deviation. BW, body weight; CA, chronological age; BA, bone age; TH, target height; PAH, predicted adult height; FH, final height.

Contrary to other studies except for one (65), the girls from our study did not exceed their PAH. The finding was unexpected since PAH was calculated before GnRHa therapy which should even improve PAH. Only our study used a rather novel BoneExpert software to calculate PAH, while other methodologies were used by other research groups, therefore the comparison of our cohort with other groups may not be accurate. In fact, PAH in our cohort was estimated at 2.8-10.3 cm higher as compared to other groups. Of note, only 42% of patients had PAH calculated according to bone age determined by the BoneXpert software, while in other patients, bone age was determined by radiologists. However, the difference between FH and TH was similar when we calculated it separately for PAH based on BoneXpert and radiologist-determined bone age.

Height outcome comparison of various cohorts of untreated girls including ours is shown in **Table 9**. Girls included in this comparison had either slowly progressive CPP or were older at onset or diagnosis. Chronological age, as well as bone age at diagnosis in girls from our study, was more advanced compared to other studies, whereas the onset of menarche was earlier, which could have influenced a higher difference between FH and TH as compared to other studies. Final heights were comparable between studies while the difference between TH and FH varied. We have to take into consideration that girls included in this comparison had slowly, milder or older onset of CPP, therefore they could have reached their target height even if they haven’t received treatment.

**Table 9:** Comparison of height outcomes in untreated girls with iCPP from different studies.

	n	CA [years]	BA [years]	Menarche [age]	TH [cm]	FH [cm]	TH-FH [cm]
Our study	15	8.75	11.01	9.50	163.5	161	-1.77
Bar et al. (67)	20	5.6	8.4	10,.5	163	161.4	-2.4
Palmert et al. (68)	16	5.5	7.9	11	164	165.5	1.5
Brauner et al. (69)	15	7.9	9.4	10.4	161	162	1
Leger et al. (57)	17	7.4	9.2	11.9	161.3	160.7	0.7
Allali et al. (70)	52	8	9.1		159.3	156.5	-2.8
Kletter and Kelch (71)	66	7.6	10.1		161.2	161.5	0.3

Values are presented as average. CA,chronological age; BA,bone age; TH,target height; FH,final height.

The most important known factors positively related to FH are younger age at onset of puberty and initiation of treatment, a shorter interval between diagnosis and onset of treatment, a longer duration of treatment, less advanced BA at diagnosis, elevated height SDS at diagnosis, greater TH, elevated height SDS and height at the cessation of treatment (72, 73).

Elevated height SDS at diagnosis is related to better height outcomes according to previous studies on girls with CPP (55, 72), which was also a finding of our study. In univariable analysis, height SDS at diagnosis was positively associated with FH SDS, yet it wasn’t revealed as a predicting factor in multiple stepwise regression, probably because its association with another variable was stronger.

More advanced puberty at diagnosis is related to worse height outcome (74) which can reflect in more advanced bone age, more advanced Tanner stage, higher basal LH concentrations as well as higher BMI due to growth spurt. Even though bone age advancement was not a predicting factor in our model, the predicted adult height, which reflects bone age, was positively associated with height outcome. Other studies similarly suggest that low PAH is negatively associated with the attainment of adult height (74, 75). Tanner stage of puberty in iCPP girls was not revealed as a predicting factor in our model and there was no significant difference in FH according to Tanner stage of puberty progression. Such an insignificant result is likely due a little variation in Tanner stage in our cohort, as only one girl had a B4 stage of breast development at diagnosis, while all the others were diagnosed at earlier stages, B2 or B3. Basal LH concentration was also a predictor for height outcome in our model. Higher values of basal LH can indicate a prolonged activation of HPG axis and therefore more advanced puberty (39). It suggests not only the importance of efficient suppression of the HPG axis for better height outcome but also the importance of early diagnosis and treatment initiation. Similarly, BMI SDS, another predictor in our study, could be related to advanced puberty at diagnosis. Previous studies demonstrated better height outcomes in children with early diagnosis, early age and shorter delay in the start of treatment (46, 73). Timely diagnosis of CPP and proper selection of patients that need GnRHa therapy is, therefore, crucial to obtaining normal FH.

Prolonged GnRHa therapy can compromise FH according to early studies (72, 74). Nevertheless, a higher height and height SDS at the cessation of treatment are known positive predictors for height outcome (72, 73), which was similarly demonstrated in our model.

Finally, inherited genetic potential as reflected in calculated TH significantly influences FH also in CPP patients (74), including our cohort.

Our study has several limitations. The most important limitation is its retrospective nature with the consequence that decisions for treatment were based on an individualized clinical decision, and not on any randomization protocol. Another limitation is that two different methods were used for bone age determination, including manual and software-based determinations. While all patients had body weight adjusted triptorelin dosing, two different triptorelin preparations were used: monthly depot before July 2007 and 3-monthly depot afterward, which is also a study limitation.

## Conclusion

Children with nCPP reached lower FH compared to the iCPP group. The group of treated iCPP and nCPP children gained 7 cm more in comparison to untreated children from diagnosis of CPP, yet there was no difference in the FH despite a 2.5-year difference in age at menarche, which resulted from individualized clinical decisions. Body weight-titrated triptorelin was effective in attaining TH and normal FH. Factors that influence FH should be considered in individualized decision-making. The most important factors that can be influenced are timely diagnosis and therapy, height at treatment cessation and prudent selection of children that can benefit from treatment.

## Data availability statement

The original contributions presented in the study are included in the article/supplementary material. Further inquiries can be directed to the corresponding author.

## Ethics statement

The studies involving human participants were reviewed and approved by Republic of Slovenia National Medical Ethics Committee (number.: 0120- 141/2017-4, KME 48/04/17, date: 8.6.2017). Written informed consent to participate in this study was provided by the participants’ legal guardian/next of kin.

## Author contributions

TK, ML, MAS, and JŠO contributed to conception and design of the study. NO, NA, TK an ML organized the database. JŽ performed the statistical analysis. TK drafted the manuscript. MA and ML wrote sections of the manuscript. TB critically reviewed the manuscript. All authors contributed to the article and approved the submitted version.

## Funding

This work received financial support from the Slovenian Research Agency (research core funding No. P3-0343).

## Acknowledgments

We are grateful to all patients and their families for their kind participation in this study. We thank the endocrinologists and nurses from the Dept. of pediatric endocrinology, diabetes and metabolism for their clinical expertise that facilitated data acquisition. We particularly thank Dr. Nina Bratanic for setting up the bases for this study by creating the clinical path for CPP management at our center.

## Conflict of interest

The authors declare that the research was conducted in the absence of any commercial or financial relationships that could be construed as a potential conflict of interest.

## Publisher’s note

All claims expressed in this article are solely those of the authors and do not necessarily represent those of their affiliated organizations, or those of the publisher, the editors and the reviewers. Any product that may be evaluated in this article, or claim that may be made by its manufacturer, is not guaranteed or endorsed by the publisher.
